# Application of a new multi-element integrated teaching mode based on bite-sized teaching, flipped classroom, and MOOC in clinical teaching of obstetrics and gynaecology

**DOI:** 10.1186/s12909-023-04494-9

**Published:** 2023-11-01

**Authors:** Wenyan Liao, Jun He, Chunfen Yang, Shuo Qi, Guodong Chen, Chengming Ding

**Affiliations:** 1 The First Affiliated Hospital, Department of Gynaecology and Obstetrics, Hengyang Medical School, University of South China, Hengyang, 421001 Hunan China; 2 The Nanhua Affiliated Hospital, Hengyang Medical School, University of South China, Hengyang, 421001 Hunan China; 3 The First Affiliated Hospital, Department of Hepatopancreatobiliary Surgery, Hengyang Medical School, University of South China, NO.69, Chuanshan Road, Hengyang, 421001 Hunan China

**Keywords:** Bite-sized teaching, Flipped classroom teaching mode, MOOC teaching mode, Obstetrics and gynaecology, Teaching mode

## Abstract

**Context:**

Effective clinical medical student education includes attention to teaching approaches. This study assessed the impact of a new multi-element teaching mode that utilizes Bite-Sized Teaching, flipped classroom, and MOOC on learner perception in an Obstetrics and gynaecology clerkship.

**Methods:**

A Two-stage crossover design study was conducted of a multi-element teaching mode compared to traditional teaching mode in an academic year. Participants included Ninety-six medical students practicing obstetrics and gynecology in our hospital, randomly divided into two groups respectively underwent multi-element teaching mode and traditional teaching mode. After each semester, a final test (including theoretical and clinical practical test) was conducted.When an academic year was completed, post intervention survey assessed learner perceptions of the intervention.

**Result:**

In order to comprehensively test students’ performance after study, we take theoretical and practical examinations. The theoretical examination mainly tests students’ grasp of basic knowledge points, while the practical examination focuses on the examination of students’ diagnosis and treatment of diseases. There were statistically significant differences both in the theoretical and clinical practical scores between the new multi-element integrated teaching mode and the traditional teaching mode, specifically as follows: In the end of first semester, the theoretical scores of the two groups were respective 43.75 ± 3.42 vs. 42.07 ± 2.90, and clinic practical test scores were respective 44.93 ± 2.42 vs. 43.37 ± 2.52; In the end of second semester, the theoretical scores of the two groups were respective 44.30 ± 2.69 vs. 42.25 ± 3.39, and clinic practical test scores were respective 43.79 ± 2.25 vs. 41.93 ± 2.80.(p < 0.05). The results of questionnaires demonstrated that 80.21% of the students showed preference for the new multi-element integrated teaching mode comparing to traditional teaching methods.

**Conclusion:**

The new multi-element integrated teaching mode is well accepted by the students and can improve the students’ mastery of knowledge, and can improve the students’ clinical comprehensive ability. The new multi-element integrated teaching mode is shown more preference than traditional teaching mode in the teaching of Obstetrics and Gynaecology. Further long term study is needed carried out to consolidate our conclusion. The new multi-element integrated teaching mode may have positive effects on clinical teaching of Obstetrics and Gynaecology.

**Supplementary Information:**

The online version contains supplementary material available at 10.1186/s12909-023-04494-9.

## Introduction

How to adopt efficient teaching methods is particularly important in improving the students’ clinical medical talents. Traditional teaching tends to be “teacher-centered”, Many studies show that traditional teaching methods have many disadvantages, such as: teachers attending classes and students passively accepting lessons with less interaction and feedback [1]; students have less motivation to learn, less enthusiastic and less effect [2]. Therefore, it is necessary and important to explore and study new teaching approach to meet this challenge.

Recently, some teaching methods that place more emphasis on interaction and clinical reasoning than traditional teaching modes have become increasingly popular. Flipped classroom has been widely adopted in the field of medical education, and it is considered helping students achieve better results in their learning [3]. In this mode, the order of classroom teaching and students’ self-study is reversed: students prepare for class by reading and/or watching pre-recorded information and lessons. Class time is spent applying new knowledge in interactive activities, such as problem solving and case discussions [4]. Massive Open line Course (MOOC) provides great convenience for students to study independently. On the MOOCs platform, learners can test themselves and interact with other learners by taking mini-tests in class after learning. It meets the requirements of free, independent, in-depth and extensive learning of medical students [5]. The combination of the flipped classroom, MOOC has been reported that it has good feasibility, acceptability and effectiveness in some fields of education [6, 7]. However, Mooc and flipped classroom mainly focus on mastering theoretical knowledge, and it is relatively weak to strengthen the practical operation of medical students. Due to the strong operability of obstetrics and gynaecology, it is very important for the treatment of emergency and critical illness. The emergence of Bite-Sized Teaching makes up for this deficiency to a large extent. Bite-Sized teaching method is one such teaching model, which uses brief, high impact e-learning video to manage cognitive load, applies multimedia principles, and promotes student’s engagement [8, 9], it focuses on a certain disease, and the micro video can help students to personally understand the characteristics and the process of the disease. Thus Bite-Sized Teaching can greatly deepen the students’ grasp of the treatment of the disease. Moreover, Bite-Sized Teaching has many advantages, such as a short teaching time, situational resource composition, strong pertinence [10]. It is reported that Bite-Sized Teaching could improve the learning efficiency and independent learning capability of medical students in Thoracic Surgery [11], and Bite-Sized Teaching are considered an effective instructional method in Health Professions Education [12], moreover, studies showed that video-enhanced problem-based learning used during the introduction of the case and formative assessment activities improved student engagement and contributed positively to the discussions and their understanding [13, 14].If Bite-Sized Teaching can be used in obstetrics teaching, it is believed that it will greatly benefit the improvement of students’ practical ability.

Therefore, we proposed a new multi-element integrated teaching mode based on flipped classroom, MOOC and Bite-Sized Teaching. But the new multi-element integrated teaching mode is not widely used in medical education yet. The influence of the flipped classroom based on MOOC and Bite-Sized Teaching in the field of obstetrics and gynaecology has not been studied. This paper intends to explore the application of the new multi-element integrated teaching mode in students teaching for obstetrics and gynaecology. The current study aims to compare traditional teaching method and the new multi-element integrated teaching mode of teaching for medical students of obstetrics and gynaecology. Objectives are to evaluate the effectiveness and students’ perception of the new multi-element integrated teaching mode comparing traditional teaching mode.

## Method

### Subjects and study setting

Our subjects were 96 medical students practicing obstetrics and gynaecology in our hospital from December 2020 to December 2021. Written informed consent was obtained from all students. (We referred to “students practicing obstetrics and gynecology” as medical students on clinical rotations on obstetrics and gynaecology during their third year of undergraduate medical training). We used the traditional teaching method and a new multi-element integrated teaching mode based on flipped classroom, MOOC and Bite-Sized Teaching mode during the students rotating in obstetrics and gynaecology. Our study was approved by The Ethics Committee of the First Affiliated Hospital of University of South China (NO. NHFY2021011608).

### The teaching process

1 For both semesters, each module of teaching content included 2 parts (theoretical lessons and clinic practical lessons.)

**Fig. 1:**
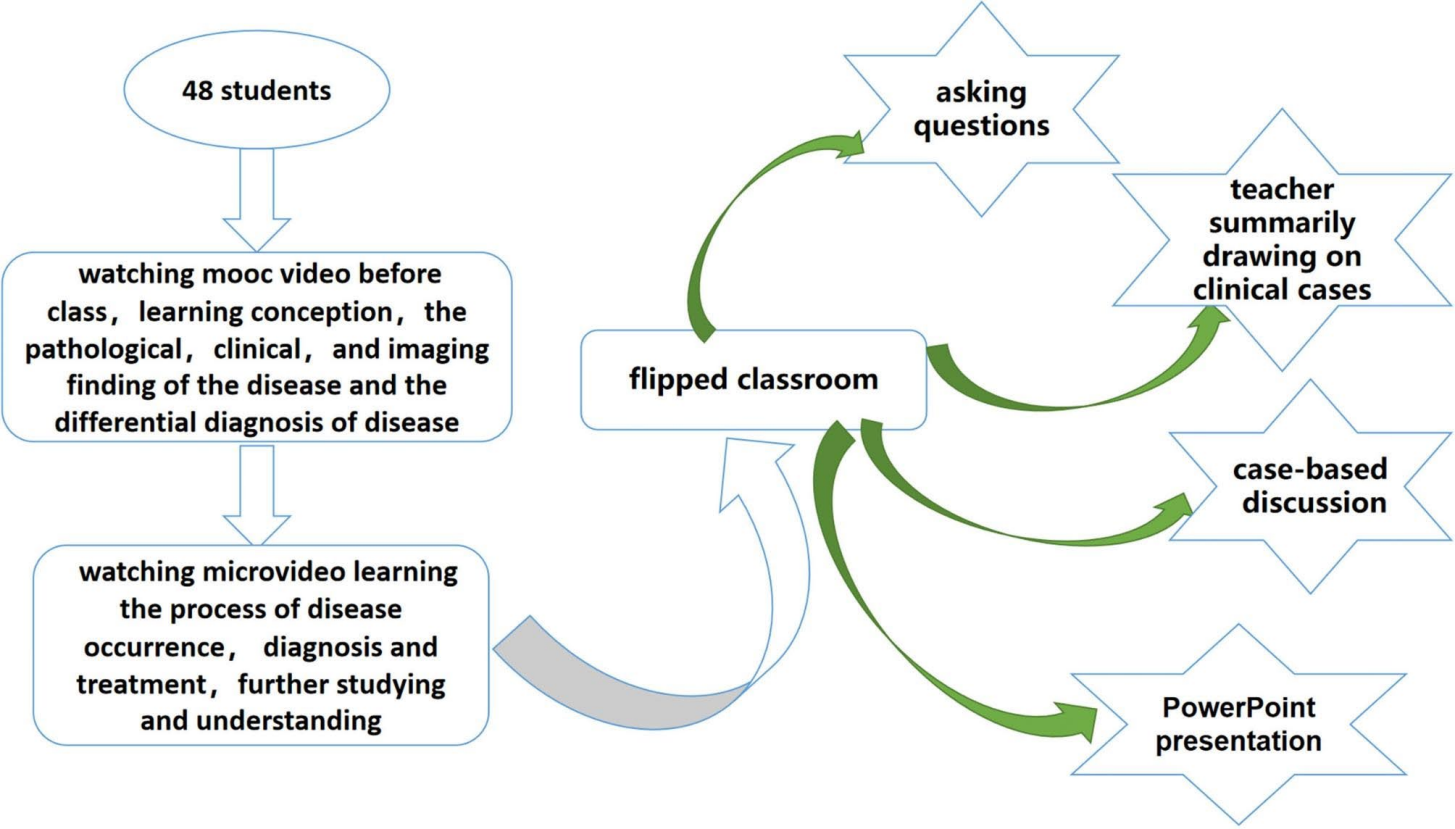
Shows the process of the new multi-element integrated teaching mode based on flipped classroom, MOOC and Bite-Sized Teaching

2 The new multi-element integrated teaching mode: Before the classroom session, students spent their spare time watching MOOC and microvideo which were made by the teacher in advance, were advised to consult more literature, and were divided into small groups to prepare a PowerPoint presentation for the class discussion. In the classroom session, teacher adopted the flipped classroom model, first, a ten-minute presentation of each group to review the main points from MOOC and Bite-Sized Teaching. Then students put forward the problems that they could not solve, each group discussed and proposed answers. For challenging questions, the teacher answered questions and summed up the learning content. Finally, teachers took students to the department of obstetrics and gynaecology in our hospital to practice related cases. Traditional teaching methods: such as small classes, lectures and clinical rounds were used. The new multi-element integrated teaching mode based on flipped classroom, MOOC and Bite-Sized Teaching was shown in Fig. 1.

### Study design

**Fig. 2:**
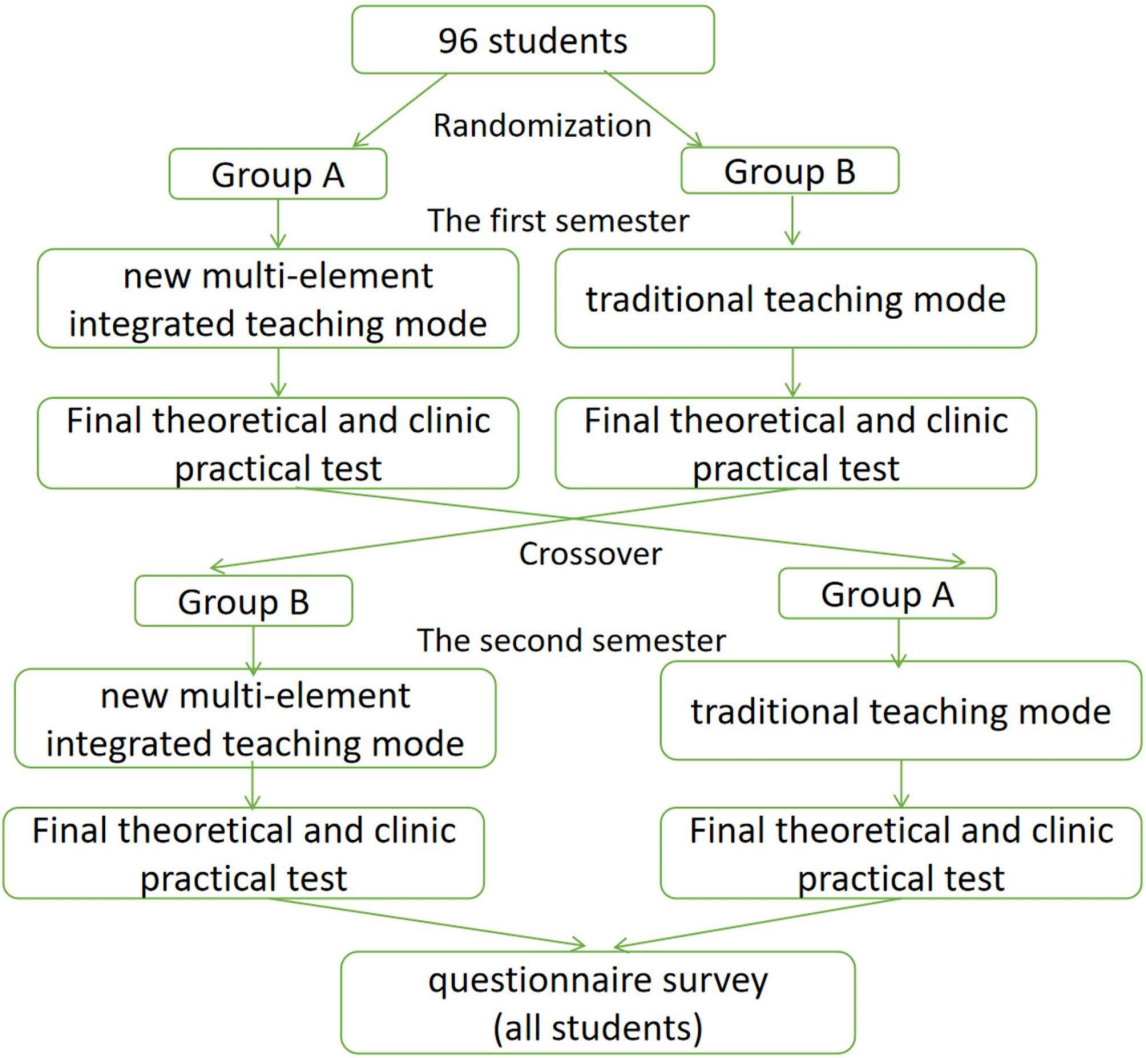
Study flow chart

This was a randomized cross-over study. The students were randomly divided into group A and group B, forty-eight students in each group. There were no statistical differences in the age, gender, and scores of school performance between the two groups (Table 1). In the first semester (From March 1st to June 1st, 2021), group A adopted the new multi-element integrated teaching mode based on flipped classroom, MOOC and Bite-Sized Teaching for their didactic curriculum A (We divided all the didactic curriculum of 1 academic year about obstetrics and gynaecology into A part and B part), while group B adopted traditional teaching mode for their didactic curriculum (A) During the second semester (From September 1st to December 1st, 2021), the teaching modes were switched between the two groups (group B adopted the new multi-element integrated teaching mode for their didactic curriculum B, while group A adopted traditional teaching mode for their didactic curriculum B). The course duration of the theoretical and practical was the same (90 min), theoretical course once a week and practical course once every two weeks. There was no repetition of course content in the two semesters to group A or group B. After each semester (The first semester on June 19th and the second semester on December 18th), a final theoretical and clinic practical test was conducted. When two semesters were completed, on December 20th, a questionnaire survey was conducted to compare and analyze the two groups of students’ independent thinking ability, critical thinking ability, teamwork ability, communication and expression ability, learning interest, learning efficiency, problem-solving ability and practical operation ability, knowledge understanding and mastery, as well as teaching method preferences (Fig. 2).

**Table 1:** Comparison of the basic situation of two groups of students

	Age($\overline{X}$±s)	School performance($\overline{X}$±s)	Gender(The male to female ratio)
Group A	21.77 ± 0.77	83.62 ± 3.37	20:28
Group B	21.52 ± 0.64	83.08 ± 3.34	20:28
t	1.706	0.7811	
p	0.091	0.436	p>0.05

### Assessment of subjective teaching effect

To assess student Subjective attitudes and experience with the new teaching mode as part of the student didactic curriculum, we designed and per-formed a cross-sectional survey of all students in our program. To do this, we conducted a focus group of students who had participated in the new teaching mode as learner during their rotation. We then created a 10-question item survey focused on learner attitudes towards the new teaching mode and its components and experiences with the new teaching mode during rotation, such as participants’ opinion about the effect of the new teaching mode to students’ ability to think independently, critical thinking ability, teamwork ability, communication and expression ability, learning interest, learning efficiency, problem-solving ability, clinical practical operation ability, ability of knowledge understanding and mastery, and preference degree of the new teaching mode. Survey was administered to all 96 students via an online survey tool.

### Assessment of knowledge acquisition

To assess impact on learning, we conducted a controlled study comparing knowledge acquisition with the new teaching mode compared to traditional teaching methods as a control. At the end of semester we created 100-question item theoretical examination and 10-question item clinic practical examination that focused on student knowledge acquisition towards the new teaching mode during their rotation, including definition, clinical manifestation, diagnosis and differential diagnosis and clinical treatment of common and frequently occurring diseases in obstetrics and gynecology. Examinations were administered to all 96 students via in-person examination.

### Data analysis

A descriptive analysis of the sample examined and an internal consistency analysis was performed through the use of the SPSS (Statistical Package for Social Science) program version 25.0. At the end of each semester, there was a theoretical test and a clinical practical test that covered all course contents. At the end of academic year, students should complete a survey to assess their experiences during exposing into the two modes. The survey was delivered on the last day of the academic year after the test. The survey included the students’ opinions of independent thinking ability, critical thinking ability, teamwork ability, communication and expression ability, learning interest, learning efficiency, problem-solving ability and practical operation ability, knowledge understanding and mastery, as well as teaching method preferences. As the results of survey, the differences between the two teaching modes were compared by using a χ2 test. The test scores were analysed by means of analysis of variance with a two-phase cross-over design and T test, and p < 0.05 was considered statistically significant.

## Results

### Subjective teaching effect

We handed out ninety-six questionnaires, recovered ninety-six, and the recovery rate was 100%. The new multi-element integrated teaching mode was well-appraised. Our data indicated that more than 80% of the students agreed that the new multi-element integrated teaching mode was beneficial to their improvement in many aspects of abilities, including the ability of independent thinking, critical thinking, teamwork, communication and expression. Then, more than 70% of the students thought that the new multi-element integrated teaching mode enhanced their learning efficiency, inaddition, the new teaching mode was good for their solving problem and practical operation ability, as well as knowledge understanding and mastery ability, and also stimulated their interest in learning. Last but not least, 80.21% of students showed preference for the new multi-element integrated teaching mode comparing to traditional teaching methods.(Table 2).

**Table 2:** Themes from teaching mode survey collected from 96 students

Themes	Number of the new teaching mode	Number of traditional teaching mode	χ^2^	p
Which teaching mode can stimulate interest in learning	73(76.04%)	23(23.96%)	52.083	0.000
Which teaching mode can improve your independent thinking ability	82(85.41%)	14(14.59%)	96.333	0.000
Which teaching mode can improve your critical thinking ability	84(87.50%)	12(12.50%)	108.000	0.000
Which teaching mode can improve your teamwork ability	78(81.25%)	18(18.75%)	75.000	0.000
Which teaching mode can improve your communication and expression ability	81(84.37%)	15(15.63%)	90.750	0.000
Which teaching mode can improve your learning efficiency	75(78.12%)	21(21.88%)	60.750	0.000
Which teaching mode can improve your ability of solving problem	80(83.33%)	16(16.67%)	85.333	0.000
Which teaching mode can improve your practical operation ability	68(70.83%)	28(29.17%)	33.333	0.000
Which teaching mode can improve your ability of knowledge understanding and mastery	71(73.96%)	25(26.04%)	44.083	0.000
Which teaching mode do you prefer	77(80.21%)	19(19.79%)	70.083	0.000

### Knowledge acquisition

**Fig. 3:**
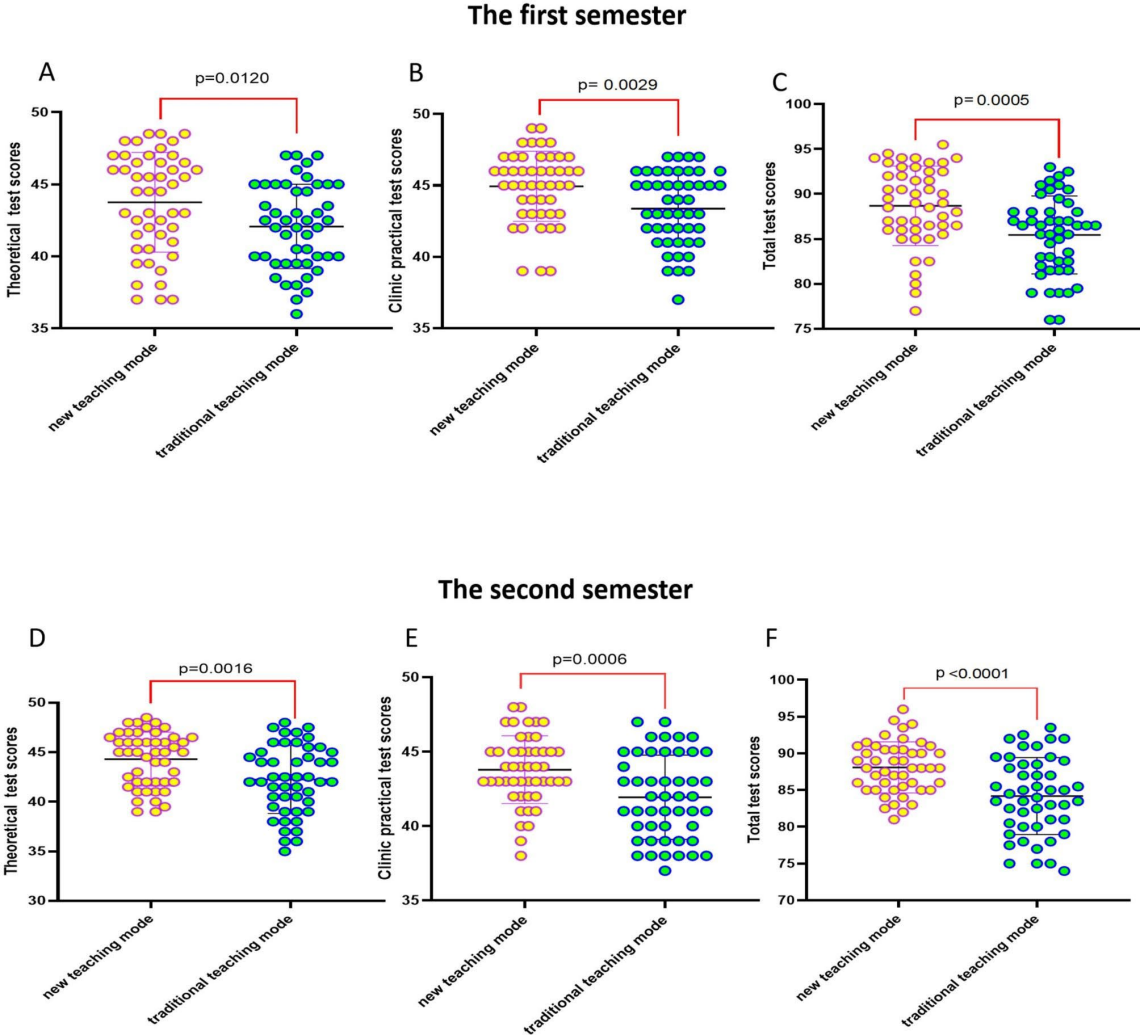
Comparison of test Results of student Between the Two teaching mode **(A-C)** In the first semester, the theoretical, clinical practical scores and total scores between the two teaching mode **(D-F)** In the second semester, the theoretical, clinical practical scores and total scores between the two teaching mode

To assess knowledge acquisition, the scores of theoretical, clinic practical test scores and total scores of all participants are shown in Fig. 3. In the first semester, the average theoretical test score of the group which under the new multi-element integrated teaching mode was 43.75 ± 3.42, the average of clinic practical test score was 44.93 ± 2.42, and the total score was 88.68 ± 4.37. While the average theoretical test score of the group which under the traditional teaching mode was 42.07 ± 2.90, the average of clinic practical test score was 43.37 ± 2.52, and the total score was 85.44 ± 4.29. Moreover, in the second semester, when students switching teaching method, the average theoretical test scores, as well as average clinic practical test scores and total scores of the group which under the new multi-element integrated teaching mode were also significantly higher than those in the traditional mode group (44.30 ± 2.69 vs. 42.25 ± 3.39, 43.79 ± 2.25 vs. 41.93 ± 2.80, 88.09 ± 3.43 vs. 84.18 ± 5.18, respectively).(Table 3). The variance analysis showed that the new multi-element integrated teaching mode has a statistically significant difference in the impact of students’ performance (Table 4).

**Table 3:** Comparison of average scores between the two teaching modes in each semester($\overline{{X}}$±s)

Test scores	The first semester				The second semester			
	Group A (Traditional teaching mode)	Group B (new teaching mode)	t	p	Group A (Traditional teaching mode)	Group B (new teaching mode)	t	p
Theoretical test scores	43.75 ± 3.42	42.07 ± 2.90	2.562	0.0120	42.25 ± 3.39	44.30 ± 2.69	3.247	0.0016
Clinic practical test scores	44.93 ± 2.42	43.37 ± 2.52	3.060	0.0029	41.93 ± 2.80	43.79 ± 2.25	3.535	0.0006
Total scores	88.68 ± 4.37	85.44 ± 4.29	3.621	0.0005	84.18 ± 5.18	88.09 ± 3.43	4.306	< 0.0001

**Table 4:** Comparative analysis of test scores between different factors

	($\overline{{X}}$±s)	F	p
The new teaching mode	88.39 ± 3.94	35.229	0.000*
Traditional teaching mode	84.81 ± 4.80		
The first semester	87.06 ± 4.62	2.377	0.127
The second semester	86.14 ± 4.81		

## Discussion

Clinical teaching of obstetrics and gynaecology has always been a difficult and important topic of medical education. Currently, there is no universally acknowledged and effective teaching method in clinical teaching of obstetrics and gynaecology. Our study found that the new multi-element integrated teaching mode based on Bite-Sized Teaching, Flipped Classroom, and MOOC could improve subjective teaching effect, such as students’ ability to think independently, critical thinking ability, teamwork ability, communication and expression ability, learning interest, learning efficiency, problem-solving ability, clinical practical operation ability, ability of knowledge understanding and mastery, preference degree, and knowledge acquisition. Our current data indicated that this new multi-element integrated teaching mode may have good prospects of application. The new multi-element integrated teaching mode contains Bite-Sized Teaching, Flipped Classroom, and MOOC. There are some studies about the application of any of this three teaching methods in medical education. Such as Al-Mugheed K et al. and Huang Z et al. found the flipped classroom method and MOOC method could improve the students’ overall mean scores of nurse students and ophthalmic skills training students [15, 16]. But the depth and breadth of knowledge mastery of teaching method with single flipped classroom method or MOOC is very limited because of lack of integration of multiple teaching methods. Bite-sized learning is an instructional method that utilizes brief, focused learning units. Theoretically the integration of multiple teaching methods can make up the shortcomings of any one teaching method, and will have better effect. Such as MOOC and Bite-Sized Teaching were reported that may be promising instructional strategy in medical education [9, 17, 18]. Since flipped classroom requires students to self-study the learning contents in advance, the MOOC and Bite-Sized Teaching are ideally applicable to this kind of demand, which can be accessed through the Internet to facilitate students’ self-study and online self-management .

There were just a few studies about the integration of MOOC, Bite-Sized Teaching with flipped classroom in education, such as Guiying Liu et al. found that integration of MOOC, Bite-Sized Teaching with flipped classroom in teaching of sanitary chemistry greatly improved the learning effect of students, stimulates their enthusiasm and initiative in learning, cultivates their independent thinking ability [19]; Lin Liu et al. proposed that integration of MOOC, Bite-Sized Teaching with flipped classroom in teaching of test signal analysis and processing course could enhance the students’ awareness of independent learning, thus improving the teaching effect [20].However, there was no papers to study the effect of this integrated teaching method in clinical practice, especially the clinical teaching of obstetrics and gynecology. In our study, we tried to organically combine MOOC, Bite-Sized Teaching with flipped classroom to form a new multi-element integrated teaching mode, then we studied the effect of this new integrated teaching mode in subjective teaching effect and knowledge acquisition of obstetrics and gynecology teaching process. This teaching mode is conducive to cultivating students’ innovative ability and helping teachers to realize their personalized teaching mode and teach students in accordance with their aptitude. Moreover, students exhibit great satisfaction and a high level of participation in the multi-element integrated teaching mode based on MOOC, Bite-Sized Teaching with flipped classroom. Our study proved that the new multi-element integrated teaching mode could improve students’ ability of grasping theoretical knowledge and operational skills of obstetrics and gynaecology, and also improve their comprehensive clinical ability to a certain extent, which are conducive for the students to keeping up with the rapid development of obstetrics and gynaecology, and lay a solid foundation for the medical students to become qualified obstetricians and gynecologists in the future.

At the same time, this study also emphasized the problems of the new multi-element integrated teaching mode based on flipped classroom, MOOC and Bite-Sized Teaching in teaching, such as the production process of high-quality videos often takes a lot of time and cost, including the collection of clinical case information and clinical data, and protecting patients’ privacy when teaching and real scenes is also an important factor in video design [21]. In addition, the current evaluation system about the new multi-element integrated teaching mode based on flipped classroom, MOOC and Bite-Sized Teaching is not good enough to achieve a comprehensive evaluation, therefore, a long-term evaluation mechanism should be studied and established for follow-up in the future.

## Conclusion

The new multi-element integrated teaching mode based on Bite-Sized Teaching, flipped classroom, and MOOC can improve the effectiveness of teaching. This new multi-element integrated teaching mode can make up for the inadequacy of traditional teaching mode. In today’s medical development, this new multi-element integrated teaching mode is worth promoting in the future medical education, and we believe that this teaching mode can cultivate more excellent doctors.

### Electronic supplementary material

Below is the link to the electronic supplementary material.

Supplementary Material 1

Supplementary Material 2

## Data Availability

The datasets generated and/or analyzed during the current study are not publicly available because they contain the patients’ personal information, but are available from the corresponding author on reasonable request.

## Declarations

**Ethics approval and consent to participate**.

Our study was approved by the Ethics Committee of the First Affiliated Hospital of University of South China and been performed in accordance with the Declaration of Helsinki. All methods were performed in accordance with the relevant guidelines and regulations. Furthermore, informed consent was obtained from all participants before the study.

**Consent for publication**.
